# Relationship between Cognitive Dysfunction and Age-Related Variability in Oxidative Markers in Isolated Mitochondria of Alzheimer’s Disease Transgenic Mouse Brains

**DOI:** 10.3390/biomedicines10020281

**Published:** 2022-01-26

**Authors:** Naoki Yoshida, Yugo Kato, Hirokatsu Takatsu, Koji Fukui

**Affiliations:** 1 Molecular Cell Biology Laboratory, Department of Systems Engineering and Science, Graduate School of Engineering and Science, Shibaura Institute of Technology, Fukasaku 307, Minuma-ku, Saitama 337-8570, Japan; mf16068@shibaura-it.ac.jp; 2 Molecular Cell Biology Laboratory, Department of Functional Control Systems, Graduate School of Engineering and Science, Shibaura Institute of Technology, Fukasaku 307, Minuma-ku, Saitama 337-8570, Japan; nb19102@shibaura-it.ac.jp; 3 Department of Medical Technology, Faculty of Health Sciences, Kyorin University, Shimorenjaku 5-4-1, Mitaka, Tokyo 181-8612, Japan; takatsu@ks.kyorin-u.ac.jp

**Keywords:** Alzheimer’s disease, reactive oxygen species, oxidative stress, cognitive impairment, mitochondria

## Abstract

Many neurodegenerative disorders, including Alzheimer’s disease (AD), are strongly associated with the accumulation of oxidative damage. Transgenic animal models are commonly used to elucidate the pathogenic mechanism of AD. Beta amyloid (Aβ) and tau hyperphosphorylation are very famous hallmarks of AD and well-studied, but the relationship between mitochondrial dysfunction and the onset and progression of AD requires further elucidation. In this study we used transgenic mice (the strain name is 5xFAD) at three different ages (3, 6, and 20 months old) as an AD model. Cognitive impairment in AD mice occurred in an age-dependent manner. Aβ1-40 expression significantly increased in an age-dependent manner in all brain regions with or without AD, and Aβ1-42 expression in the hippocampus increased at a young age. In a Western blot analysis using isolated mitochondria from three brain regions (cerebral cortex, cerebellum, and hippocampus), NMNAT-3 expression in the hippocampi of aged AD mice was significantly lower than that of young AD mice. SOD-2 expression in the hippocampi of AD mice was lower than for the age-matched controls. However, 3-NT expression in the hippocampi of AD mice was higher than for the age-matched controls. NQO-1 expression in the cerebral cortex of AD mice was higher than for the age-matched controls at every age that we examined. However, hippocampal NQO-1 expression in 6-month-old AD mice was significantly lower than in 3-month-old AD mice. These results indicate that oxidative stress in the hippocampi of AD mice is high compared to other brain regions and may induce mitochondrial dysfunction via oxidative damage. Protection of mitochondria from oxidative damage may be important to maintain cognitive function.

## 1. Introduction

Alzheimer’s disease (AD) is a severe irreversible brain disorder. The number of patients suffering from it in Japan is currently higher than 3 million, and the rate of its increase there is the fastest in the world [1]. The most important symptom of AD is dementia via dysfunction of cholinergic neurons [2]; in particular, neuronal cell death occurs frequently in the hippocampal region [3]. Donepezil hydrochloride, an AD therapeutic, acts as an inhibitor of acetylcholine esterase [4], but because treatment with donepezil does not stop AD progression [5], the development of novel therapeutic agents is necessary.

To elucidate the mechanisms of AD onset and progression, researchers have used many experimental models, such as fruit flies [6], Caenorhabditis elegans [7], and monkeys [8]. The most frequently used animal models are transgenic mice, and many kinds have been developed [9]. AD research focuses on two major neuropathological features, namely, the accumulation of amyloid-beta (Aβ) [10] and tau hyperphosphorylation [11], but their relative contributions have not been determined.

Reactive oxygen species (ROS) such as superoxide anion radicals are mainly produced in the mitochondrial electron transport chain [12,13]. Superoxide is reduced to hydroxyl radicals in several steps and attacks cell membranes. Because oxygen is essential for life, ROS production cannot be eliminated in aerobic organisms. The accumulation of oxidative products such as lipid hydroperoxides is associated with many serious diseases such as aging, cancer, cardiovascular disease, arteriosclerosis, and high blood pressure [13,14]. In particular, the brain is rich in polyunsaturated fatty acids and consumes a large volume of oxygen compared to other organs; thus, it is easily affected by ROS. Recently, many reports have noted a relationship between ROS and AD [15]. ROS accelerates Aβ production, and Aβ plaques release ROS, resulting in neuronal cell death [16]. Our previous study found Aβ deposits in the hippocampal CA1 region of rats with vitamin E deficiency, normally aged rats, and young rats exposed to high oxygen [17]. These animals showed significant oxidation, especially in the brain, compared to normal young rats [17].

To prevent AD onset and progression, identification of early changes in the brain is necessary, and many researchers have searched for suitable markers. In the early stage of AD, axonal degenerative effects, such as shrinkage and beading, appear in the brain [18,19]. These phenomena are also seen in other neurodegenerative disorders such as Huntington’s disease [20] and multiple sclerosis [21]. In our previous study, treatment with a low concentration of hydrogen peroxide induces axonal degeneration [22]. In this condition, calcium homeostasis is disrupted, and mitochondrial oxidation is induced [23]. These results indicate that mitochondrial dysfunction via oxidation is strongly related to the onset and progression of AD. To further our understanding of this relationship, in this study we used AD-transgenic mice (5xFAD) and isolated mitochondria at different ages to measure ROS-related protein expression.

## 2. Materials and Methods

### 2.1. Animals

All animal experiments were approved by the Animal Protection and Ethics Committee of Shibaura Institute of Technology (Approval number #15001), and 5xFAD transgenic mice (#008730, MMRRC034848, B6.Cg-Tg (APPSwFlLon, PSEN1*M146L*L286V) 6799Vas/Mmjax, Alias/5XFAD) from The Jackson Laboratory (Bar Harbor, ME, USA) and self-bred prior to use in these experiments. In the present study, 3-, 6- and 20-month-old AD-transgenic mice were used. C57BL/6 Ncr male mice of the same age were obtained from Sankyo Labo Service Corp. Inc. (Tokyo, Japan) and used as a control group. All mice were maintained in conditions of controlled temperature (22 ± 2 °C) and a 12 h light/dark cycle, and were provided with free access to food and water. Food consisted of normal diet pellets (Labo MR Stock) purchased from Nosan Corp. (Kanagawa, Japan). Cognition and motor function were assessed using tests as described below. Following assessments, the mice were euthanized, and brain samples (cerebral cortex (Cortex), cerebellum (Cer), and hippocampus (Hip)) were collected for analysis. All other chemical agents were obtained from FUJIFILM Wako Pure Chemical Corp. (Osaka, Japan).

### 2.2. Behavioral Assessment

#### 2.2.1. Morris Water Maze

Cognitive function was assessed using a Morris water maze apparatus [24,25]. The maze apparatus (140 cm in diameter and 45 cm in height) consists of a pool constructed of acrylic resin. The bottom of the pool was divided into four quadrants by lines and was set up with four different visible marks positioned around the pool. A submerged platform was placed in the center of one quadrant. The water temperature of the pool was maintained at 22 ± 2 °C. Before starting the cognitive performance trials, the animals were acclimated to the pool. by being allowed to swim freely for 60 s in the absence of a platform, and to handling by the experimenter over a 3-day period. The cognitive trials were performed four times per day and continued for five consecutive days. All trials were performed at the same time of day, and were carried out every 3 h (starting at 9:00, 12:00, 15:00, and 18:00). We performed a total of 20 trials per mouse. The platform was maintained in the same location of the pool for all trials. The escape latency (time to reach the goal), swimming distance, swimming speed, and the proportion of time spent swimming in the quadrant containing the platform were measured using the ANY-maze software (ver. 4.98; Stoelting Co., Wood Dale, IL, USA).

#### 2.2.2. Rota-Rod Test

The rota-rod (Muromachi Kikai Co., Ltd., Tokyo, Japan) test was used to assess coordinated movement ability, as described previously with some modifications [26]. The rod was accelerated from 5 to 50 rpm over a duration of 120 s. The latency to the time to fall was measured.

### 2.3. Mitochondria Isolation

Mitochondria were isolated from the cerebral cortex, cerebellum and hippocampus of each mouse using a self-made buffer (2.5 M sucrose, 5 mM 2-[4-(2-hydroxyethyl)-1-piperazinyl] ethanesulfonic acid (HEPES), 1 mM ethylenediaminetetraacetic acid (EDTA), pH 7.2). After sonication, the samples were centrifuged at 500× *g* for 10 min at 4 °C. The supernatant was centrifuged at 10,000× *g* for 10 min at 4 °C. The precipitate was diluted in lysis buffer and mixed with 5× sodium dodecyl sulfate (SDS) buffer.

### 2.4. Western Blotting

All samples except isolated mitochondria samples were homogenized in lysis buffer and used in Western blotting as described previously [27], with some modifications. Sample lysates were centrifuged, and protein contents were determined using the Bradford assay (Bio-Rad protein assay, #500-0006JA, Bio-Rad Laboratories, Inc., Hercules, CA, USA) according to the manufacturer’s protocol. Protein extracts (brain homogenate samples of 10 and 50 μg, respectively) were separated on 10, 12, and 15% SDS-polyacrylamide gels and transferred to polyvinylidene difluoride (PVDF) transfer membranes (Immobilon; Merck KGaA, Darmstadt, Germany). The PVDF membranes were washed and incubated in blocking solution (Tris-HCl-buffered saline, pH 7.6 (TBS), containing 0.1% Tween 20 and 2% non-fat skim milk) for 1 h at room temperature. The membranes were washed in TBS containing 0.1% Tween 20, and then treated with each primary antibody (anti-β-amyloid (1-40), mouse monoclonal antibody (4H308), #GTX17420, GeneTex Inc., Los Angeles, CA, USA; anti-amyloid beta 42 rabbit polyclonal antibody, #PA3-16761 Thermo Fisher Scientific Inc., Waltham, MA, USA; anti-tau [TAU-5] mouse monoclonal antibody, #ab80579, Abcam Inc., Cambridge, UK; anti-tau (phosphor-S262) rabbit polyclonal antibody, #ab131354 Abcam Inc.; anti-brain-derived neurotropic factor (BDNF) (N-20) rabbit polyclonal antibody, #sc-546 SANTA CRUZ BIOTECHNOLOGY (SCBT) Inc., Dallas, TX, USA; anti-nerve growth factor (NGF) (H-20) rabbit polyclonal antibody, #sc-548, SCBT Inc.; anti-tropomyosin receptor kinase A (TrkA) (763) rabbit polyclonal antibody, #sc-118, SCBT Inc.; anti-TrkB (H-181) rabbit polyclonal antibody, #sc-8316, SCBT Inc.; rabbit polyclonal anti-COX-IV mitochondrial loading control antibody, #ab16056, Abcam plc.; mouse monoclonal [2A12] anti-3-nitrotyrosine antibody (3-NT), #ab52309, Abcam plc.; mouse monoclonal anti-cytochrome C [37BA11] antibody, #ab110325, Abcam plc.; goat polyclonal anti-nicotinamide mononucleotide adenylyl transferase (NMNAT-3) antibody, #ab121030, Abcam plc.; rabbit polyclonal anti-nicotine adenine dinucleotide phosphate (NAD(P)H) quinone oxidoreductase-1 (NQO-1) antibody, #bs-2184R, Bioss Antibodies Inc., Woburn, MA, USA; rabbit polyclonal anti-superoxide dismutase-2 (SOD-2) antibody, #bs-1080R, Bioss Antibodies Inc.) overnight at 4 °C. Anti-mouse, -rabbit or -goat IgG horseradish peroxidase-conjugated antibodies (Promega Corp., Madison, WI, USA) were used as secondary antibodies at 1:4000 dilution for 1 h at room temperature. All Western blotting experiments were performed at least three times. All chemiluminescent signals were generated by incubation with the detection reagents (Immobilon; Merck KGaA) according to the manufacturer’s protocol. For normalization of the bands for each protein, the membranes were reprobed with anti-α-tubulin rabbit monoclonal antibody (#2125, Cell Signaling Technology Inc., Danvers, MA, USA). The relative intensities were determined using LAS-3000 (FUJIFILM Corp., Tokyo, Japan). Expression ratios were calculated by dividing each protein value by that of α-tubulin using ImageJ software (National Institutes of Health, Bethesda, MD, USA).

### 2.5. Statistical Analysis

Data are expressed as means ± standard error (SE). They were analyzed using GraphPad Prism 9.2.0 (GraphPad Software, San Diego, CA, USA); *p* values of less than 0.05 were considered statistically significant. The detailed statistical methods are described in the individual figure captions.

## 3. Results

**Figure 1:**
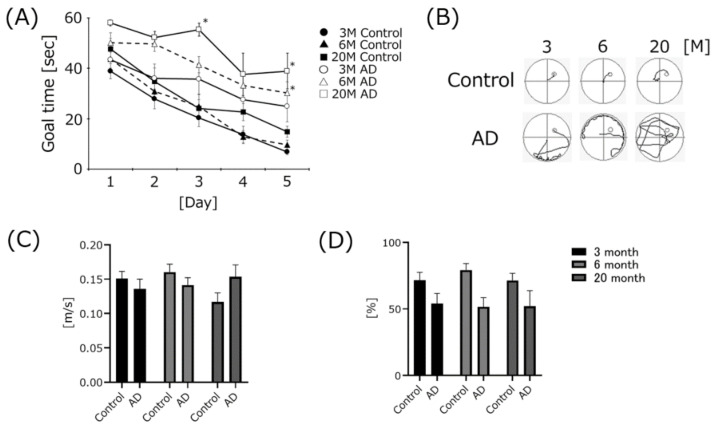
Differences in cognitive function between control and Alzheimer’s disease (AD) transgenic mice depending on age. The time to goal (escape latency) in the Morris water maze test is shown in panel (**A**). The swimming trajectory and swimming speed are shown in panels (**B**,**C**). The ratio of staying time in the platform quadrant is shown in panel (**D**). Three different ages of AD mice (3 M, 6 M, 20 M) were used (3 M AD, *n* = 10; 6 M AD, *n* = 10; 20 M AD, *n* = 5). Age-matched C57BL/6 mice were used as a control group (3 M control, *n* = 15; 6 M control, *n* = 15; 20 M control, *n* = 10). * *p* < 0.05, vs. the age-matched control group. The data are shown as means ± SE. Statistical analyses of goal time were performed using two-way analysis of variance. Statistical analysis of goal time among all group was performed using the two-way analysis of variance. Statistical analyses of the goal time of each day, swimming speed, and the ratio of staying time in the platform quadrant were performed using the Tukey–Kramer method.

The results of the Morris water maze test are shown in Figure 1. The goal time gradually decreased for all mouse groups. The average goal times on the final trial day for 6- and 20-month-old AD mice were significantly higher than those of age-matched control groups (Figure 1A). The swimming trajectories of the AD mice showed that their swimming distances were remarkably longer than those of age-matched controls (Figure 1B). However, no significant differences in the swimming speeds among all mouse groups were noted (Figure 1C). The ratio of staying time in the platform area was higher for the control mice compared to the age-matched AD mice (Figure 1D). However, no significant differences were found for any mouse groups.

**Figure 2:**
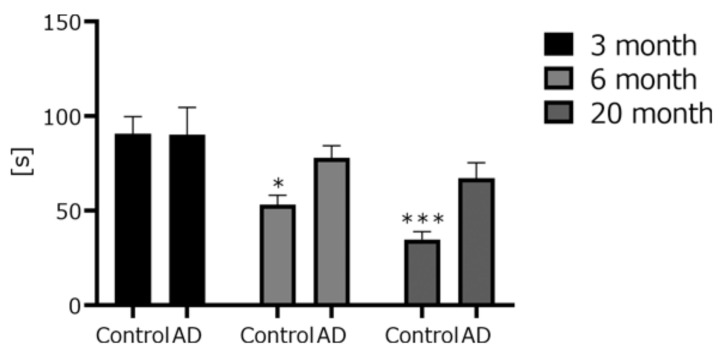
Time to fall in the rota-rod test. Three different ages of AD mice (3 M AD, *n* = 8; 6 M AD, *n* = 8; 20 M AD, *n* = 8) and age-matched C57BL/6 mice (3 M control, *n* = 8; 6 M control, *n* = 8; 20 M control, *n* = 5) were used. * *p* < 0.05 *** *p* < 0.001 vs. 3 M control. The data are shown as means ± SE. Comparisons were performed using the Tukey–Kramer method.

The coordination abilities were measured using a rota-rod apparatus (Figure 2). The time to fall for control and AD mice gradually decreased in an age-dependent manner. The time to fall for young mice did not differ between AD and control mice. However, the time to fall for 6- and 20-month-old control mice tended to decrease compared to the age-matched AD mice.

**Figure 3:**
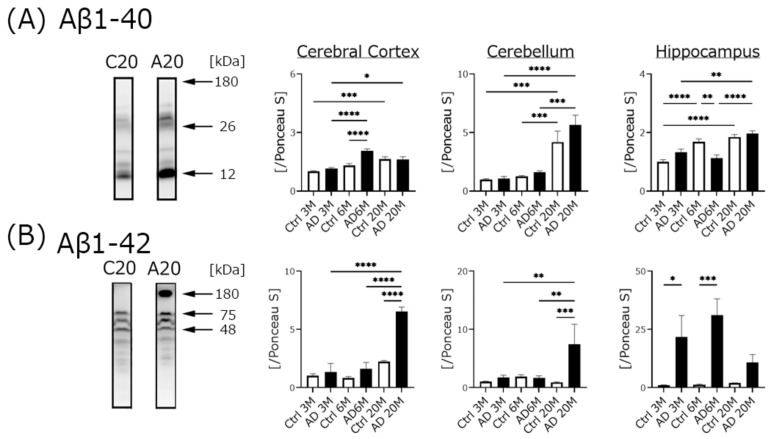
Changes in the levels of Aβ protein according to Western blot and immunohistochemical analyses. Western blotting experiments were performed using three different ages (3, 6, and 20 months) and three different brain regions (cerebral cortex, Cortex; cerebellum, Cer; hippocampus, Hip). Total protein expression in all bands was calculated for Aβ1-40 analysis (**A**). For Aβ1-42 analysis, the high-molecular-weight band (amyloid fibrils) was used for calculation (**B**). The ratio of each protein band intensity to Ponceau S intensity is shown in panels (**A**) and (**B**), with the ratios of each brain region of 3 M control samples set to 1. Black columns show 3 M, 6 M, and 20 M AD transgenic mice (3 M AD, *n* = 9; 6 M AD, *n* = 8; 20 M AD, *n* = 5), and white columns show age-matched C57BL/6 mice (3 M control, *n* = 10; 6 M control, *n* = 10; 20 M control, *n* = 10). Asterisks show significant differences (* *p* < 0.05, ** *p* < 0.01, *** *p* < 0.001, **** *p* < 0.0001). The data are shown as means ± SE. Comparisons were performed using the Tukey–Kramer method; 6 M AD and control mice were used.

We measured Aβ protein expression using Western blotting, and measured band intensities for Aβ1-40 expression (Figure 3A). Aβ1-40 protein expression in all regions in AD and control mice gradually increased in an age-dependent manner. A band of high molecular size (fibril formation), representing Aβ1-42 was seen, and the band intensity was calculated (Figure 3B). Aβ1-42 expression in the hippocampus of AD mice dramatically increased compared to age-matched controls. The ratio of the hippocampal score for Aβ1-42 expression in the AD mice was higher than the score in the other two brain regions.

**Figure 4:**
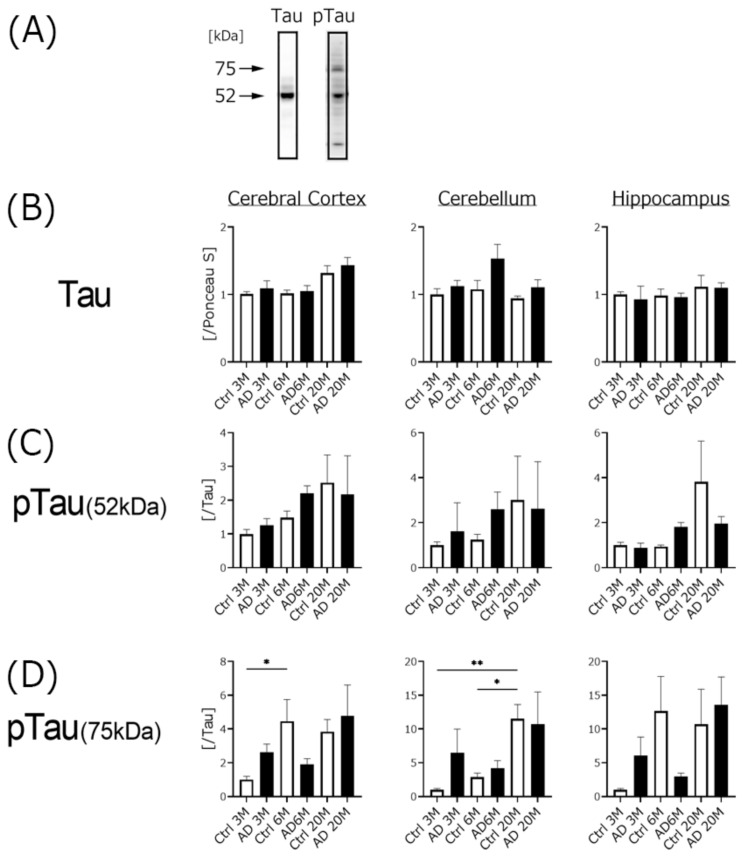
Western blotting analysis of the levels of tau and phospho6-tau expression in the brains of three different ages of AD mice. All experiments were performed using three different brain regions (cerebral cortex, Cortex; cerebellum, Cre; hippocampus, Hip). Western blotting images are shown in panel (**A**). Tau protein expression was calculated and shown in panel (**B**). Phospho-tau protein expression was calculated using two different band sizes (52 (**C**) and 75 kDa (**D**)). The ratio of each phospho-tau band intensity to Ponceau S intensity is shown, with the ratios of each brain region of 3 M control samples set to 1. Black columns show 3 M, 6 M, and 20 M AD mice (3 M AD, *n* = 10; 6 M AD, *n* = 10; 20 M AD, *n* = 5), and white columns show age-matched C57BL/6 mice (3 M control, *n* = 10; 6 M control, *n* = 10; 20 M control, *n* = 5). Asterisks show significant differences (* *p* < 0.05, ** *p* < 0.01). The data are shown as means ± SE. Comparisons were performed using the Tukey–Kramer method.

We used 5xFAD mice in this experiment, in which no mutation at the site of tau phosphorylation is present. To analyze the relationship between Aβ accumulation and the ratio of tau protein including the phosphorylated form in AD mice, we analyzed tau and phospho6-tau expression in the three different brain regions with Western blotting (Figure 4).

No differences in tau expression were seen among any samples. Two bands of different sizes representing phospho-tau (52 and 75 kDa) were detected and calculated. Each phospho6-tau expression was nominally increased (but not significant) in AD and control mice in an age-dependent manner. However, phospho6-tau (75 kDa) in the 6-month-old AD mice tended to be lower than that in the 3-month-old AD mice.

**Figure 5:**
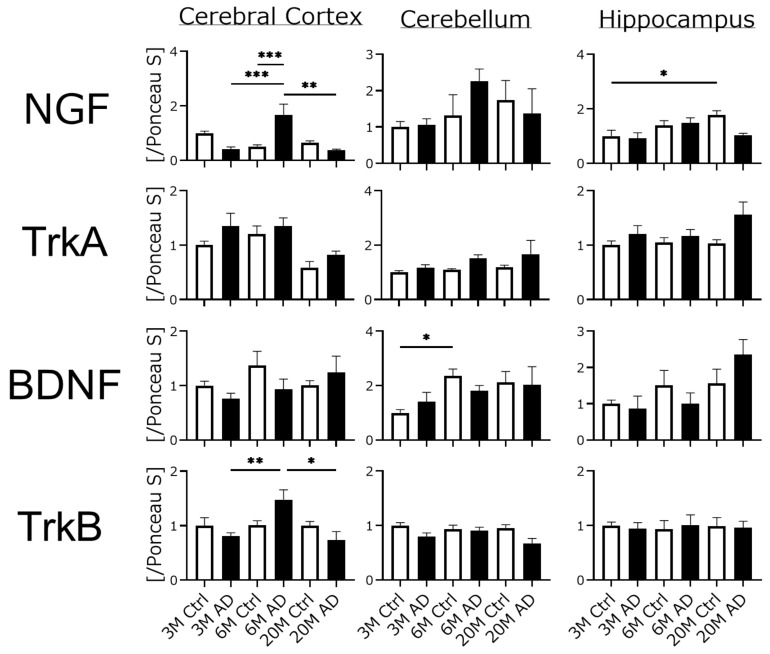
Western blotting analysis of the levels of neurotrophic-factor-related proteins in the brains of three different ages of AD transgenic mice. All experiments were performed using three different brain regions (cerebral cortex, Cortex; cerebellum, Cre; hippocampus, Hip). The ratio of each protein band intensity to Ponceau S intensity is shown, with ratios of each brain regions of 3 M control samples set to 1. Black columns show 3 M, 6 M, and 20 M AD mice (3 M AD, *n* = 8; 6 M AD, *n* = 8; 20 M AD, *n* = 4), and white columns show age-matched C57BL/6 mice (3 M control, *n* = 8; 6 M control, *n* = 8; 20 M control, *n* = 8). Asterisks show significant differences (* *p* < 0.05, ** *p* < 0.01, *** *p* < 0.001). The data are shown as means ± SE. Comparisons were performed using the Tukey–Kramer method.

To clarify the relationship between cognitive dysfunction in AD mice and neuronal function, we assessed the expression of neurotrophic factors and their receptor proteins (Figure 5). However, the expression ratios of NGF, BDNF, and their receptors did not differ in any brain regions of either mouse group, except for NGF and TrkB expressions in the cortex region, and parts of cerebellum and hippocampus.

**Figure 6:**
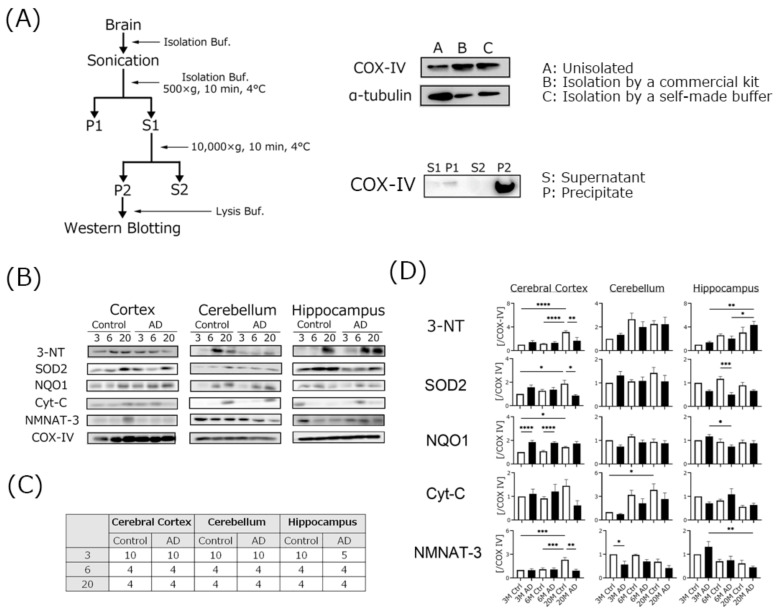
Western blotting analysis of the levels of each protein in isolated mitochondria of brains of three different ages of AD-transgenic mice. All experiments were performed using three different brain regions (cerebral cortex, Cortex; cerebellum, Cer; hippocampus, Hip). The mitochondrial isolation method is shown in panel (**A**). Western blotting images are shown in panel (**B**). The number of each sample is shown in panel (**C**). The ratios of each protein band intensity to COX-IV intensity are shown, with ratios of each brain region in 3 M control samples set to 1 (**D**). Black columns show 3 M, 6 M, and 20 M AD mice. White columns show age-matched control mice. Asterisks show significant differences (* *p* < 0.05, ** *p* < 0.01, *** *p* < 0.001, **** *p* < 0.0001). The data are shown as means ± SE. Comparisons were performed using the Tukey–Kramer method.

We isolated mitochondria and measured protein expression using Western blotting. First, we checked the quality of our mitochondria isolation technique using our self-made buffer (Figure 6A). After isolation, we checked COX-IV expression using Western blotting, and found that the COX-IV band was very clear. These results suggest that our self-made buffer and isolation method has the same separation ability as the commercial kit. Hippocampal 3-NT expression in the AD and control mice gradually increased in an age-dependent manner. The 3-NT expression level in the hippocampus of the AD mice was higher than that in the age-matched controls. NQO-1 expression levels in the cerebral cortex of the AD mice were higher than those in the age-matched controls. However, the hippocampal SOD-2 expression level in AD mice was lower than those in the age-matched controls. NMNAT-3 expression tended to be lower in 20-month-old AD mice compared to the age-matched controls of all brain regions.

## 4. Discussion

### 4.1. AD Transgenic Mice Developed Cognitive Dysfunction, but Swimming Speed Did Not Change

To clarify the relationship between senescence and cognitive function in AD transgenic mice, we assessed their cognitive function using the Morris water maze task at three different ages. The goal time gradually decreased for all mouse groups with or without AD. The goal time of the final trial day gradually decreased in an age-dependent manner in both mouse groups. These results showed that cognitive function was gradually impaired by aging with or without AD and are consistent with our previous study [24]. The goal times on the final trial day for 6- and 20-month-old AD mice were significantly impaired compared to the age-matched controls. The goal time on the final trial day for the 3-month-old AD mice was nominally impaired (but not significantly) compared to the age-matched controls. The swimming distance of the AD mice was longer than that of the age-matched controls. These results showed that the AD mice developed cognitive dysfunction even at a young age, and the difference in the goal time between the controls and the AD mice at the same age gradually widened.

To clarify that these differences were not simply due to motor dysfunction, we measured swimming speed. We found no significant differences among the mouse groups. Additionally, we measured coordination ability using the rota-rod test. The fall time gradually decreased in an age-dependent manner in mice with or without AD. The fall time for the 20-month-old control group was significantly faster than that of the 3-month-old group. These results, using two different apparatuses, showed that AD mice did not induce much motor dysfunction and coordination disorder. Even during normal breeding, we found no particular difference in gait or movement in the AD mice. We found that the cognitive function of the AD mice simply deteriorated at a younger age. However, the fall time in the rota-rod test for the AD mice tended to be nominally longer (but not significantly so) compared to that of the age-matched controls. The reason is likely due to the difference in body weight. The fall time in the rota-rod test tends to be body-weight-dependent. In our previous study, obese mice fell faster than age-matched controls, and a negative correlation between body weight and fall time was observed [28]. In this study, the mean body weights of the AD mice at each age were lower than those of the age-matched controls, despite being fed ad libitum (control 3-month-old (3 M), 22.44 g; control 6-month-old (6 M), 30.8 g; Control 20-month-old (20 M), 29.18 g; AD 3 M, 18.6 g; AD 6 M, 20.4 g; AD 20 M, 24.2 g). The difference in the fall time in the rota-rod test may be due to the difference in weight between the two groups. The reason why the body weight of the AD mice was lower than those of the age-matched controls is unknown.

### 4.2. Aβ Expression, but Not Phospho-Tau Was Significantly Increased in AD Transgenic Mice

In this experiment, we purchased AD transgenic mice, which are widely used [29,30]. Basic information on the 5xFAD strain is provided on the JAX official website (https://www.jax.org/strain/008730, accessed on 17 December 2021). This strain overexpresses both human Aβ precursor protein with the Swedish (K670N, M671L), Florida (I716V), and London (V717I) Familial Alzheimer’s Disease (FAD) mutations and human presenilin-1 harboring two FAD mutations, M146L and L286V. JAX recommends that this strain be used as an Aβ1-42-induced neurodegeneration model. To elucidate which AD-related proteins affected cognitive dysfunction in the Morris water maze trial, we examined Aβ1-40, -42, tau, and phospho-tau protein expression using Western blotting analysis.

Aβ1-40 expression in the AD mice was increased in all brain regions in an age-dependent manner. However, the rate of the increase in its expression in the AD mice, especially in the 20-month-old mice, was lower than that of Aβ1-42, and the control mice also gradually showed an increase in Aβ1-40 in every brain region in an age-dependent manner. Aβ1-42 expression in the AD mice also increased in an age-dependent manner, except in the hippocampus of the 20-month-old mice. Surprisingly, the increased rate of Aβ1-42 expression in the hippocampus was much higher than that of the other two brain regions in all AD mouse groups. Generally, the toxicity of Aβ1-42 is considered to be stronger than that of Aβ1-40 [31], and Aβ1-42 tends to aggregate [32]. Changes in the ratio of Aβ1-40 and -42 are associated with Aβ toxicity, and increased Aβ1-42 exacerbates the AD condition [33]. We selected a high-molecular-weight band (more than 180 kDa) and used it in the calculations for this study. Our results indicated that Aβ1-42 began to aggregate in the hippocampus of the AD mice from a young age. JAX explains that Aβ1-42 aggregation begins to accumulate in 1.5-month-old mice, which is broadly consistent with our results. Unfortunately, we did not examine the protein expression of both Aβ species in mice younger than 3 months. Because AD mice are self-breeding, they are expensive, time consuming, and very difficult to produce in quantity. We prioritized the creation of a 20-month-old group in this study. In the near future, we intend to conduct a similar study in AD mice younger than 1.5 months to clarify when Aβ1-42 aggregates.

We found no significant difference in tau expression in any of our samples. We calculated phospho-tau expression using two different molecular weight bands, and the expression of the 75-kDa band gradually increased in an age-dependent manner in both the AD and the control mice, except in the 6-month-old AD mice. The increased rate of 75-kDa phospho-tau expression was higher than that of the 52-kDa band, and hippocampal expression of the 75-kDa band was higher than that in the other two brain regions in both the AD and the control mice. These results indicate that cognitive impairment in AD mice is likely induced by Aβ1-42 aggregation in the hippocampus.

### 4.3. Neurotrophic Factors and Their Receptor Expression Were Unchanged in AD Transgenic Mouse Brains

Neurotrophic factors such as NGF and BDNF play an important role in the maintenance of higher brain function. The functions of neurotrophic factors include axonal elongation, neuronal survival, and neurotransmitter synthesis [34]. To clarify the effect of neurotrophic factor expression in cognitive dysfunction in AD mice, we examined the expression of NGF, BDNF, and their receptors with Western blotting. Contrary to expectation, we found no significant differences in any samples except for parts of the NGF and TrkB expressions in the cerebral cortex. In general, neurotrophic factor secretion declines gradually with age [35], but this study did not show that trend. Cholinergic neurons are severely damaged in AD mice, but NGF-releasing cells may not be significantly affected, and the rate of damage or dysfunction may vary by neuron type. Had we looked at the mRNA, a difference may have been seen or large individual differences may have been apparent. Any underlying causal factors remain unknown.

### 4.4. Mitochondrial Oxidative Damage Was Increased in AD Transgenic Mouse Brains

ROS attack many tissues and induce oxidative damage and cell death. The “free radical theory of aging” hypothesis was first proposed by Harman in 1956 [36]. The accumulation of oxidative damage is related to the onset or progression of many severe diseases, including neurodegenerative disorders and aging [13]. Oxidation levels in the brain are accelerated in AD mice and cause various symptoms [37]. Lipid hydroperoxides are one golden marker for determining oxidation. Thiobarbituric acid-reactive substances, 4-hydroxynonenal and prostaglandin F2alpha may also be measured. However, these are end products. We wanted to determine the oxidation-related markers that occur further upstream, not in the final product. We thought that there might be significant changes upstream, even if there were no significant differences in the final product. In particular, mitochondrial dysfunction is critical and plays a key role in aging and disease [38], and mitochondrial dysfunction-induced cellular calcium homeostasis deterioration is a major issue [15,39]. In our previous study, treatment with a low concentration of hydrogen peroxide induced neurite degeneration in neuroblastoma cell lines, and mitochondria accumulated in regions of neurite degeneration [40]. Excessive influx of calcium ions induces mitochondrial superoxide generation and membrane oxidation [23]. These results suggest that ROS-induced mitochondrial damage may be closely related to neuronal survival.

To determine the mitochondrial oxidation levels, we measured the expression of proteins using isolated mitochondria. SOD-2 (also known as Mn-SOD), which detoxifies superoxide, is specifically present in mitochondria. SOD-2 mutations are related to the onset of AD, Parkinson’s disease, and stroke [41]. SOD-2 expression levels, especially in the hippocampus, were nominally (but not significantly) lower compared to age-matched controls in our experiment. This result indicates that AD mice may have an imbalanced redox state. However, 3-NT expression in the hippocampi of the AD mice was higher than that in the age-matched controls. 3-NT, which is an index of nitric oxide production in cells, is synthesized from peroxynitrite and tyrosine, and peroxynitrite is synthesized from superoxide and nitric oxide [42]. 3-NT is a marker of protein modification and is related to many diseases such as arteriosclerosis and AD [43,44]. These results indicated that nitric oxide production in the hippocampus of AD mice may be relatively high compared to other brain regions of AD mice, as well as in age-matched controls.

NMNAT-3 levels were also attenuated in every region of AD mouse brains in this study. NMNAT-3 catalyzes the formation of nicotinamide adenine dinucleotide (NAD+) synthesis from nicotinamide mononucleotide and ATP, and is localized in the mitochondria [45]. NAD+ is needed for the citric acid cycle and respiratory chain in mitochondria, and the NAD+ level is an index of mitochondrial function [46]. The activation of NMNAT-3 induces mitochondrial function and has an anti-aging effect in NMNAT-3 over expression mouse model [47]. We could not demonstrate a direct interaction between oxidative stress and changes in NMNAT-3 levels in mitochondria. Low NMNAT-3 levels in AD mice indicate that mitochondrial function may be impaired by ROS. Further investigation is needed to clarify this interaction.

NQO-1 is a flavin-adenine-dinucleotide-dependent flavoprotein that serves as an oxidative stress response protein [48]. NQO-1 expression is regulated by a complex of the Kelch-like ECH-associated protein 1 (Keap 1) and the nuclear factor erythroid 2-related factor 2 (Nrf2) system [49]. The induction or knockdown of NQO-1 may be directly related to the reduction or enhancement of the oxidative stress status in cells [50]. NQO-1 expression in the cerebral cortex of the AD mice was higher than that in the age-matched controls at every age that we examined. However, hippocampal NQO-1 expression in 6-month-old AD mice was significantly lower than 3-month-old AD mice. These results suggest that the antioxidant response function, including the Keap1/Nrf2 system, was functioning in the cerebral cortex, albeit with gradual weakening. In the hippocampus, the response of defense mechanisms including the expression of antioxidant enzymes such as SOD-2 to ROS was already weakened, and as a result, oxidation including 3-NT was promoted. Oxidation may result in impaired mitochondrial function, such as by NMNAT-3, leading to mitochondrial dysfunction, and finally, to the development of cognitive dysfunction.

However, all analytical data for Western blotting of isolated mitochondria were normalized by COX-IV. COX-IV is one of the mitochondrial-specific proteins and is a subunit of cytochrome c oxidase in the mitochondrial respiratory chain. For Western blotting using isolated mitochondria, COX-IV, VDAC1/Porin, and heat shock protein 60 (HSP60) may be used as loading controls. However, care should be taken when using these proteins as loading controls. COX-IV may already be damaged in AD transgenic mice [51,52]. It is important to consider the use of multiple loading proteins for more accurate results. In that sense, this result may not be completely correct, so that this should be kept in mind for future experiments.

## 5. Conclusions

In our experiments, we found that the AD mice showed significant damage from ROS. However, the number of samples of isolated mitochondria from AD mice was limited other than for the outside of the cerebral cortex region. If mitochondria can be protected from ROS, mitochondrial function and cognitive function may be maintained. Future studies should explore compounds that can specifically exert a strong antioxidant capacity in mitochondria.

## Data Availability

All data generated or analyzed during this study are included in this published article.
